# Evidence for persistent multilocus genotypes of *Biomphalaria pfeifferi* in a natural population in Kenya, with implications for transmission of *Schistosoma mansoni*

**DOI:** 10.1186/s13071-025-06881-1

**Published:** 2025-06-21

**Authors:** Noel A. Oduor, Daniel W. Kariuki, Gerald M. Mkoji, Polycup O. Oraro, Martina R. Laidemitt, Michelle L. Steinauer, Eric S. Loker, Eric L. Agola

**Affiliations:** 1 Biochemistry Department, School of Biomedical Sciences, Jomo Kenyatta University of Agriculture and Technology, Nairobi, Kenya; 2 Center for Biotechnology Research and Development, Kenya Medical Research Institute, Nairobi, Kenya; 3 College of Osteopathic Medicine of the Pacific–Northwest, Western University of Health Sciences, Lebanon, OR USA; 4 Department of Biology, Center for Evolutionary and Theoretical Immunology, Parasite Division Museum of Southwestern Biology, University of New Mexico, Albuquerque, NM USA

**Keywords:** Multilocus Genotypes, *Biomphalaria pfeifferi*, *Schistosoma mansoni*, Red queen, Neglected tropical diseases, Self-fertilizing snails

## Abstract

**Background:**

*Biomphalaria pfeifferi*, a predominantly self-fertilizing freshwater snail, is the world’s most important intermediate host for *Schistosoma mansoni*, one of the causative agents of schistosomiasis, a neglected tropical disease affecting millions of people in sub-Saharan Africa. We sought to determine whether we could identify distinct and persistent lineages of *B. pfeifferi* within a natural stream habitat in western Kenya, indicative of their asexual descent. We also sought to determine whether infections by *S. mansoni* or other trematodes were associated with particular lineages.

**Methodology:**

Utilizing 14 microsatellite markers in a multiplex polymerase chain reaction (PCR) format, we genotyped 502 *B. pfeifferi* collected in six bimonthly (every other month) sampling times from the same locality in a single habitat (Asao Stream, western Kenya). Snails were isolated and screened for infection with *S. mansoni* and other trematodes using the shedding method followed by microscopical examination of any cercariae found.

**Results:**

We identified 26 multilocus genotypes (MLGs), that were present at two or more sampling times. Four MLGs persisted across the entire 10-month sampling period, one of which was represented by 17 individuals. These persistent lineages harbored a variety of trematode species, with *S. mansoni* being the most common. The persistent MLGs were more likely to have trematode infections than those found only at a single sampling time. Low genetic differentiation was observed between November and March (fixation index among subpopulations [F_ST_] = 0.019; *p* =  < 0.05). The highest genetic differentiation was observed between July and March (F_ST_ = 0.372; *p* =  < 0.001). Analysis of molecular variance (AMOVA) showed higher variation among individuals within sampling times (58%) than within individuals (33%), and a smaller variation (8%) was found among sampling times.

**Conclusions:**

By identifying the presence of persistent MLGs and their associations with trematode transmission, this study highlights the importance of considering *B. pfeifferi* MLGs, some of which could be resistant to infection, when developing strategies to control schistosomiasis transmission within Asao Stream and similar ecosystems across sub-Saharan Africa.

**Graphical Abstract:**

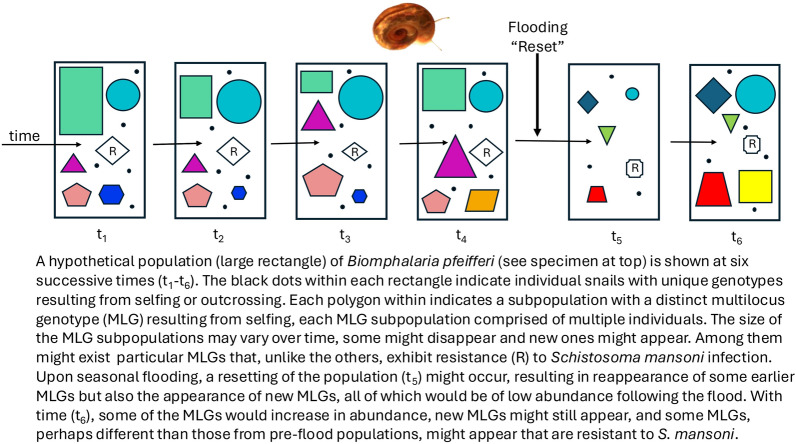

**Supplementary Information:**

The online version contains supplementary material available at 10.1186/s13071-025-06881-1.

## Background

*Biomphalaria pfeifferi*, a planorbid gastropod, is commonly found in freshwater ecosystems across sub-Saharan Africa [1]*.* It is the single most important intermediate host for *Schistosoma mansoni*, the most prevalent of *Schistosoma* species causing human intestinal schistosomiasis [2]. There are more than 231 million cases of human schistosomiasis worldwide, the majority of cases are in sub-Saharan Africa [3]. Several factors continue to facilitate the persistence of schistosomiasis in sub-Saharan Africa. These include poverty, poor sanitation, and the use of untreated water from lakes and rivers for drinking and washing. Limited access to healthcare also makes it difficult to control the disease [4]. Effective control strategies for schistosomiasis rely on a multifaceted approach, including interrupting the parasite’s life cycle. This requires an understanding of the biology, underlying genetics, and transmission potential of the intermediate freshwater snail hosts of the genus *Biomphalaria* [5].

Previous studies focusing on the population structure of *B. pfeifferi* have consistently provided strong evidence of preferential selfing associated with inbreeding, with relatively low levels of intrapopulation variation when compared with interpopulation variation [6, 7]. In some areas, where opportunities for colonization are limited, such as Oman [6], or where recent population expansions have occurred, as in Senegal [8], individual *B. pfeifferi* populations often show very low levels of genetic diversity as compared with populations where opportunities for colonization are higher, such as Madagascar [9].

These population genetics studies, though, do not directly address the issue of the extent to which, as a consequence of selfing, distinct clusters of related individuals that share multilocus genotypes (MLGs) exist within the selfing populations of *B. pfeifferi*. Importantly, can particular MLGs be identified that represent the progeny produced by self-fertilization, and do they persist over time? Although these clusters of individuals would not necessarily be expected to be genetically identical because of the possibilities of some recombination occurring during selfing, the members of a particular MLG might nonetheless share important attributes, such as being especially susceptible, or refractory, to particular parasites, including *S. mansoni* [10].

Previous studies in Senegal have in fact suggested that, in areas not previously occupied by *B. pfeifferi*, a colonization event occurred possibly originally consisting of a single *B. pfeifferi* lineage, one in this case highly susceptible to *S. mansoni* infection and that favored an outbreak of intestinal schistosomiasis [8]. This reinforces the idea that different *B. pfeifferi* MLGs exhibit varying levels of susceptibility to *S. mansoni*, possibly including some that are refractory to schistosome infection. If *S. mansoni*-resistant MLGs are present, they might limit transmission and potentially could be identified and exploited in novel snail-resistance-based control programs. The thought of using snail-immune-based resistance to *S. mansoni* has been developed using the related schistosome vector *Biomphalaria glabrata* as a model, for which genetically based resistance and inbred *S. mansoni*-resistant lines are known [11–13]. Better understanding the potential variation in susceptibility among *B. pfeifferi* MLGs holds significant value for schistosomiasis control.

For *B. pfeifferi*, if MLGs persist over time, it is also conceivable through Red Queen dynamics that local parasites might adapt to and become more numerous in locally common genotypes [14]. Such dynamics could be prevented from developing, though, because particular habitats may be dramatically altered by flooding or drought, leading to the elimination of established MLGs. The underlying reason for the existence of selfing may be most apparent in favoring rapid colonization of habitats frequently “reset” by flooding, drought, or other environmental changes [15]. As both annual and longer-term abiotic environmental changes are well-known to have major effects on populations of aquatic schistosome vector snails, including favoring both expansions and collapses of snail populations, such effects will have to be taken into account for resistance-based strategies to succeed [16–18].

Microsatellites markers were used in this study to identify MLGs. Microsatellites are powerful genetic tools because they have dense distribution in the genome, have co-dominant inheritance, are fairly simple to identify, and have high variability [19]. Their high level of variation makes them extremely useful for spotting differences within a population and between individual organisms, and they were the technology available to us at the time [20].

Environments such as Asao Stream in Kisumu County, west Kenya, which are prone to flooding and seasonal variations, offer ideal conditions to determine whether MLGs can be identified, and whether they persist over time, and thus through seasonal variations. The primary aims of this study are to determine whether MLGs within *B. pfeifferi* populations in Asao Stream can be identified and whether they persist across multiple sampling times, and to learn whether particular MLGs are either associated with or show a pattern of excluding infections with *S. mansoni* and other trematode infections.

## Methods

### Ethics statement

This project was undertaken with the ethical approval of the Kenya Medical Research Institute (KEMRI) Scientific and Ethical Review Unit (SERU), reference number KEMRI/SERU/CBRD/224/4252. The project and its methodology have also been approved by Kenya’s National Commission for Science, Technology and Innovation (NACOSTI) under the Research License 622444.

### Site of collection of *B. pfeifferi* snails and bimonthly samples

**Fig. 1:**
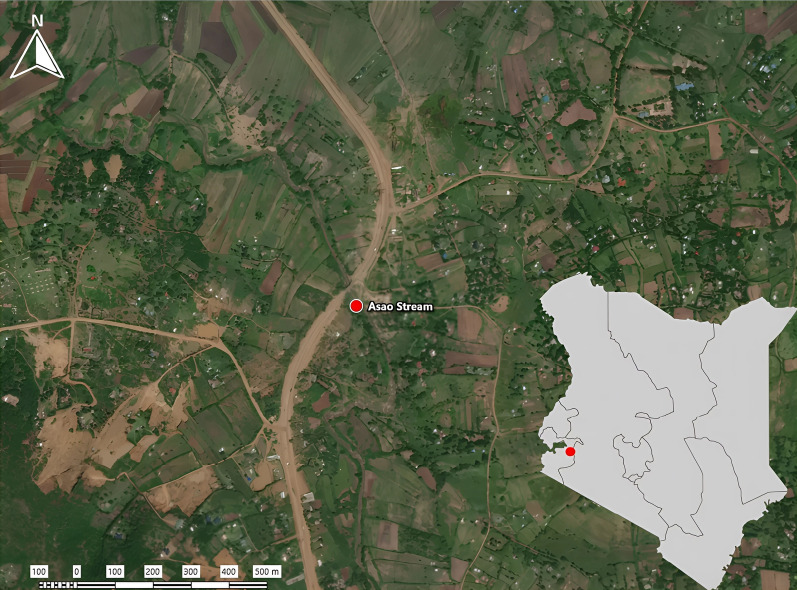
Map of Kenya showing the location of the study site, on Asao Stream. (Map was created using ExpertGPS version 9.3) [58]

Snails for this study were sourced from Asao Stream, which is located in Asao village, Nyakach district, within Kisumu County, western Kenya (Global Positioning System [GPS] coordinates −0.318086, 35.006904; Fig. 1). Asao village is located 45 km south of Kisumu City on the Kisumu–Sondu road, in western Kenya. The stream originates from the Mau escarpment and flows into Lake Victoria through Asao village within the lake basin plainlands. The area experiences hot and humid climatic conditions. Generally, the average temperature range in the region is 16 °C (at night) to 30 °C, with a bimodal rain seasonality, characterized by long rains between February and May and short rains between October and December. The average rainfall ranges between 800 and 1900 mm. However, especially considering ongoing climate change, variability in these weather patterns from year to year is to be expected. Asao Stream was first identified over 30 years ago to be a *B. pfeifferi* habitat in which *S. mansoni* transmission occurs and has since been the site of other studies of schistosomes and snails [21, 22].

*Biomphalaria pfeifferi* were collected from Asao Stream during six bimonthly (every other month) collections, from November 2018 to September 2019. In the morning hours, with the assistance of the same two experienced snail collectors, a long-handled snail scoop was used to sweep aquatic vegetation along the shoreline of Asao Stream at the same designated location (where the stream crosses under the Kisumu–Sondu road) over a 30-min collection period. This site is known to reliably contain *B. pfeifferi* and is a contact point frequented by local people and their domestic animals. All *B. pfeifferi* collected were rinsed with aged tap water and then isolated in individual wells of 24-well plates and exposed to indirect sunlight for 1–2 h between 10:00 a.m. and 2:00 p.m. to determine whether they released (shed) cercariae of any trematode species. Shedding was observed using a dissecting microscope, and then cercariae were collected with a glass pipette, placed on a glass microscope slide, stained with Lugol’s iodine, and then identified morphologically at 40× or 100× with the aid of a compound microscope. Cercariae were identified according to trematode keys [23, 24] and were broadly placed into the following groups: *Schistosoma mansoni*, strigeids, paramphistomoids, echinostomes, xiphidiocercariae, or sanguinicolids.

Asao Stream is a known significant focus of human *S. mansoni* infection [22]. The closely related rodent-transmitted species *Schistosoma rodhaini* also occurs at Asao at low prevalence, for example, accounting for 1 of 45 (2.2%) identifiable schistosome infections from *B. pfeifferi* [25]. The likelihood of *S. rodhaini* appearing among our samples is lessened further by the fact that the *B. pfeifferi* were isolated for shedding at a time of day largely coinciding with the optimal shedding time of Kenyan *S. mansoni* (10 a.m.–2 p.m.) whereas peak shedding for Kenyan *S. rodhaini* occurs at 6 a.m., with fewer cercariae shed thereafter until evening [25]. Although we note the possibility of *S. rodhaini* occurring among our samples, as indeed it might in many African *Biomphalaria* habitats, we also feel justified in referring to the mammalian schistosome cercariae we recovered from *B. pfeifferi* as *S. mansoni* in our results below. All cercariae identified as paramphistomoids were confirmed according to Sey [26].

### DNA extraction from *Biomphalaria pfeifferi*

We employed the HotSHOT method, originally described by Truett [27], to extract DNA from the head–foot tissue of *B. pfeifferi* snails. This method offers a rapid and efficient way to isolate genetic material. Briefly, head–foot tissue was severed from each snail with a razor blade and then was transferred to an individual well of a 96-well plate using sterile techniques. A total of 75 μL HotSHOT lysis solution (composed of 25 mM NaOH and 0.2 mM disodium ethylenediaminetetraacetic acid [EDTA]) was added to each well. The plates were then vortexed to ensure thorough mixing and then centrifuged for 20 s at 16,000 ×*g*. To prevent evaporation and contamination, the plates were sealed with a silicone mat before being placed in an Eppendorf Mastercycler Systems thermocycler with a heated lid for incubation at 95 °C for 1 h. After this incubation step, the reaction was stopped by adding 75 μL of HotSHOT neutralization reagent (containing 40 mM tris(hydroxymethyl)aminomethane [Tris]–HCl; pH 5.0) to each well. The concentration of each DNA isolate was calculated by absorbance of 1 μL of the sample at 260/280 nm using a Nanodrop 2000 spectrophotometer (Thermo Fisher Scientific).

### Microsatellite typing

DNA amplification was carried out in in a multiplex polymerase chain reaction (PCR) format using two panels of microsatellites, both of which are specific to *B. pfeifferi* [6, 28–30]. Panel 1 targeted Bpf4, Bpf8, Bpf12, U2, Rg3, Bpf1, and Bpf2, while the second panel focused on Rg1, U-7, Rg4, Rg5, Bpf5, Rg8, and Rg6 (Table 1). The QIAGEN Multiplex PCR Kit was used for amplification in 14 μL reactions according to the manufacturer’s instructions. The primer concentration per reaction was at 1 μM. The concentration of DNA varied across samples with an average of 696 ng/μL.

**Table 1:** Target microsatellite loci, primer oligo sequences, approximate product length (in base pairs), fluorescent dyes, and GenBank accession numbers

Marker	Primer sequence	Amplicon size range (bp)	Fluorescent dye	Accession number
Bpf4	F: GAATTCAGTCCTTACAGCAGC R: CTTAAGCTGCGCTATCTGTGG	317–329	VIC	AF189701
Bpf8	F: GGTTCCCATAGCATACAGTGC R: GGCTTACAAAGAAACAGGCATAC	193–225	6FAM	AF189704
Bpf12	F: GACACAAAGAAAGAGATAAGCA R: GTCGACCTCCCACTCTTC	307–334	6FAM	AF189708
U-2	F: TCAATTTGCAAAGTTCAAGAGC R: GTAATCCAAAGCAACCGACTG	140–148	PET	AY425953
Rg3	F: TGACACGGCTAGTGATGAGG R: AGAACAGGGATGAACAATGG	180–182	HEX	JN205162
Bpf 1	F: TCCTATCCTTGTAACTTCTCCAC R: CGAAACCATGCAAATCAG	203–207	HEX	AF189698
Bpf 2	F: GCAGCTTCATTCAACATTCC R: AAATTAACATTTCGCTGAAACAG	139–153	VIC	AF189699
Rg1	F: TGGGCAGTTCCAATGAGG R: TGAGAAAGCCAACAAAGG	125–131	6FAM	JN205160
U-7	F: TCATGACATCTAATGGGAGAAG R: GGGCTCAGAGAAATGAATGG	222–246	6FAM	AY425954
Rg4	F: GGTCACTTTTATTTGGCAACAG R: GTGGATAGATGGGGAGTTAGGG	185–187	NED	JN205163
Rg5	F: GTCAAAATATCTGCACTGATGG R: GAAACCTGCCTTTTTACATGC	196–198	6FAM	JN205164
Bpf 5	F: TGTATGCTGACACTTAAAGAAACC R: GCTACGCCACTGCTTATGAC	175–187	HEX	AF189702
Rg8	F: GCAAGTCATTAGGAGGATGAGG R: GACACTGCGATTAGGTCAAGG	264–268	VIC	JN205167
Rg6	F: GATTTTGTCTCACGGAAACG R: GCGTGCTTATGTAGCAAAGG	233–244	PET	JN205165

6-FAM, 6-carboxyfluorescein; HEX, hexachlorofluorescein; NED, 2′-chloro-5′-fluoro-7′,8′-benzo-1,4-dichloro-6-carboxyfluorescein; PET, photoinduced electron transfer; VIC, 2′-chloro-7′-phenyl-1,4-dichloro-6-carboxyfluorescein

The thermal cycling profile included an initial denaturation step at 95 °C for 15 min, followed by 35 cycles for 30 s at 94 °C, an annealing temperature specific to each multiplex panel (52 °C for Bp1 and 55 °C for Bp2) for 60 s, an extension at 72 °C for 60 s, and a final step of 10 min at 72 °C using an Eppendorf Master cycler Systems thermocycler. Multiplexed PCR products were then diluted in *N*,*N*′-dimethylformamide with Genescan^®^–500 (LIZ 500) as an internal size standard and genotyped using an ABI3500XL automated sequencer (Applied Biosystems) and scored with GeneMaker^®^ version 3.0.1 software (Soft Genetics). All genotype calls were verified manually. MLGs are unique combinations of alleles across all loci within an organism, and they were calculated by using a rank function on strings of alleles. For snails to be considered the same MLG, they had to be identical at all microsatellite loci.

### Statistical analysis

Microsoft Excel 2016 [31], GenAlex version 6.5 software [32], and R studio-based packages poppr version 2.9.6 and Coin version 4.4 [33–36] were used to analyze data in this study. The R package poppr was utilized to determine the number of multilocus genotypes (MLGs) and the MLGs that crossed sampling times. The MLGs were calculated using 14 codominant loci (Table 1). To address potential bias arising from cloned genotypes, clone correction was done while identifying MLGs that crossed sampling times. This process removed duplicated MLGs within population strata. Phylogenetically uninformative loci (monomorphic across all individuals and loci with excessive missing data) were also eliminated. The R package coin was used to assess whether those snails identified with the most common MLG retrieved—MLG190—were found significantly more often than what we expected on the basis of chance using a permutation test. Excel was used to calculate descriptive statistics [37]. GenAlEx provides a range of analysis tools for handling genetic data [38], and was used to calculate allelic frequencies, population assignment tests, expected heterozygosity (H_e_), values of fixation index among subpopulations (F_ST_) analysis (999 permutations), the number of alleles per sampling time, percentage of polymorphic loci, analyses of molecular variance (AMOVA), and determination of MLG similarity, and a Mantel test was to assess the correlation between F_ST_ values and temporal distance (999 permutations). The unbiased estimator *f̂* of Wright’s inbreeding coefficient among subpopulations *F*_IS_ was calculated using *F*_*IS*_ = (H_e_ − H_o_)/H_e_ according to Weir and Cockerham [39]. Selfing rate known to be independent of the mutation model was estimated using the relationship *s* = 2*F*_*IS*_/(1 + *F*_*IS*_) [40, 41]. Graphs were generated using GraphPad Prism version 10.4.2 [42]

## Results

**Fig. 2:**
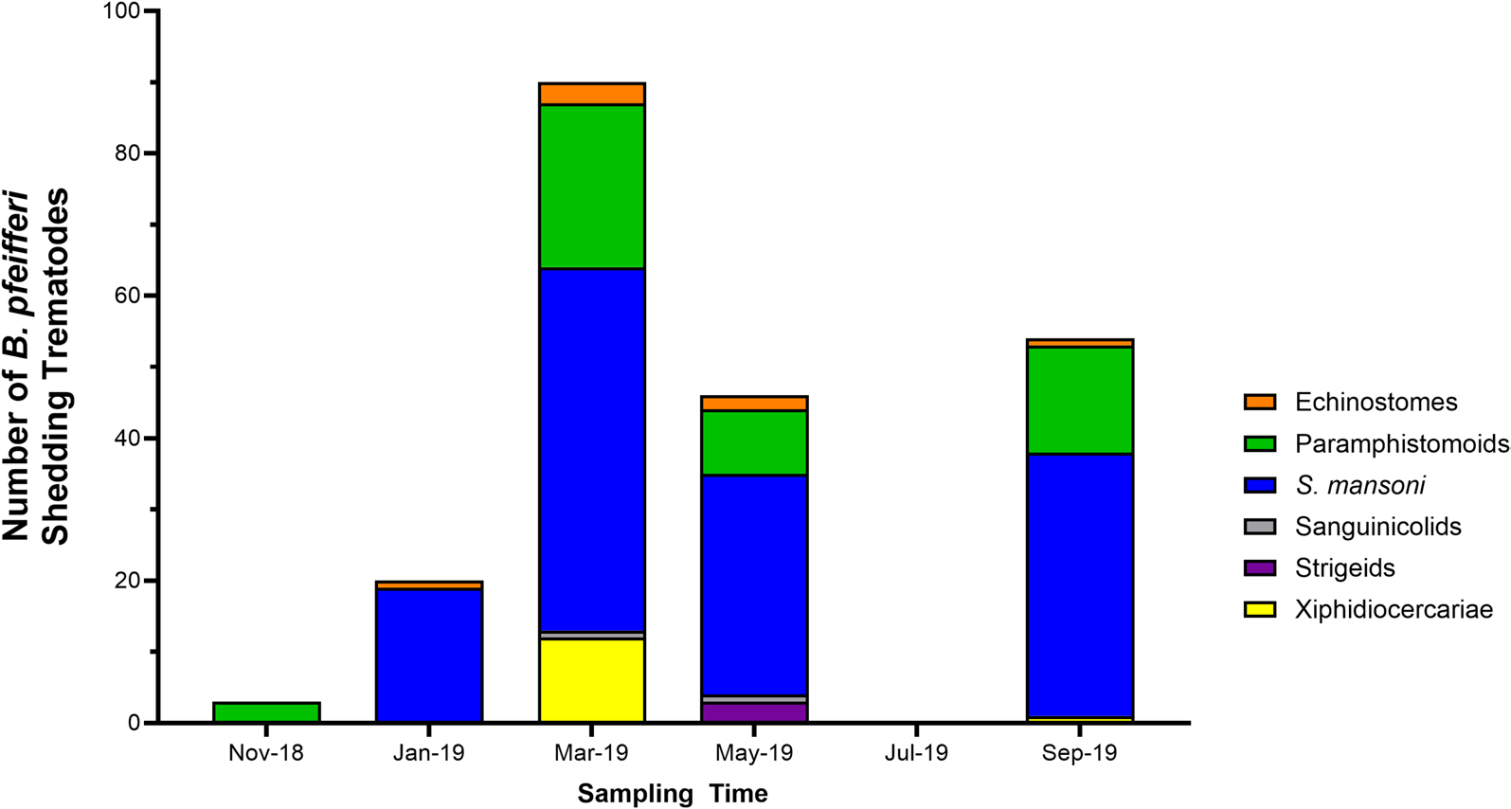
Total number of *B. pfeifferi* snails with trematode infections (determined on the basis of morphology) at each sampling time

A total of 502 *B. pfeifferi* were collected during the six bimonthly sampling times (Fig. 2; Table 2). The relative abundance of *B. pfeifferi* varied over the collection period, with the lowest values being in July 2019, when water levels were highest. At each of the six sampling points, the number of *B. pfeifferi* with or without patent (shedding cercariae) trematode infections was determined. Trematode infections were identified as being *S. mansoni*, strigeids, paramphistomoids, xiphidiocercariae, echinostomes, and sanguinicolids. While the November 2019 collection yielded the most snails (120 individuals), it also had relatively few infected snails—only 3 with paramphistomoids. March had the most infected snails (a total of 90), harboring *S. mansoni*, strigeids, paramphistomoids, xiphidiocercariae, echinostomes, and sangunicolids. July had the fewest snails [22] with no trematode infections present (Fig. 2). Of 502 snails collected, 138 were infected with *S. mansoni* (27.5%). The overall prevalence of all trematodes was 42.4% (213/502), with values indicative not only of intense transmission in Asao Stream of *S. mansoni* but also of trematodes in general.

**Table 2:** Numbers of *Biomphalaria pfeifferi* collected at each sampling time and their infection status

	Sampling time	Total *n*	Samples	*n* per sample
1	November (2018)	120	No trematodes	117
			Paramphistomoids	3
2	January (2019)	56	No trematodes	36
			*S. mansoni*	19
			Echinostomes	1
3	March (2019)	118	No trematodes	28
			*S. mansoni*	51
			Paramphistomoids	23
			Xiphidiocercariae	12
			Echinostomes	3
			Sangunicolid	1
4	May (2019)	84	No trematodes	38
			*S. mansoni*	31
			Paramphistomoids	9
			Strigeids	3
			Echinostomes	2
			Sanguinicolid	1
5	July (2019)	22	No trematodes	22
6	September (2019)	102	No trematodes	48
			*S. mansoni*	37
			Paramphistomoids	15
			Echinostomes	1
			Xiphidiocercariae	1

**Fig. 3:**
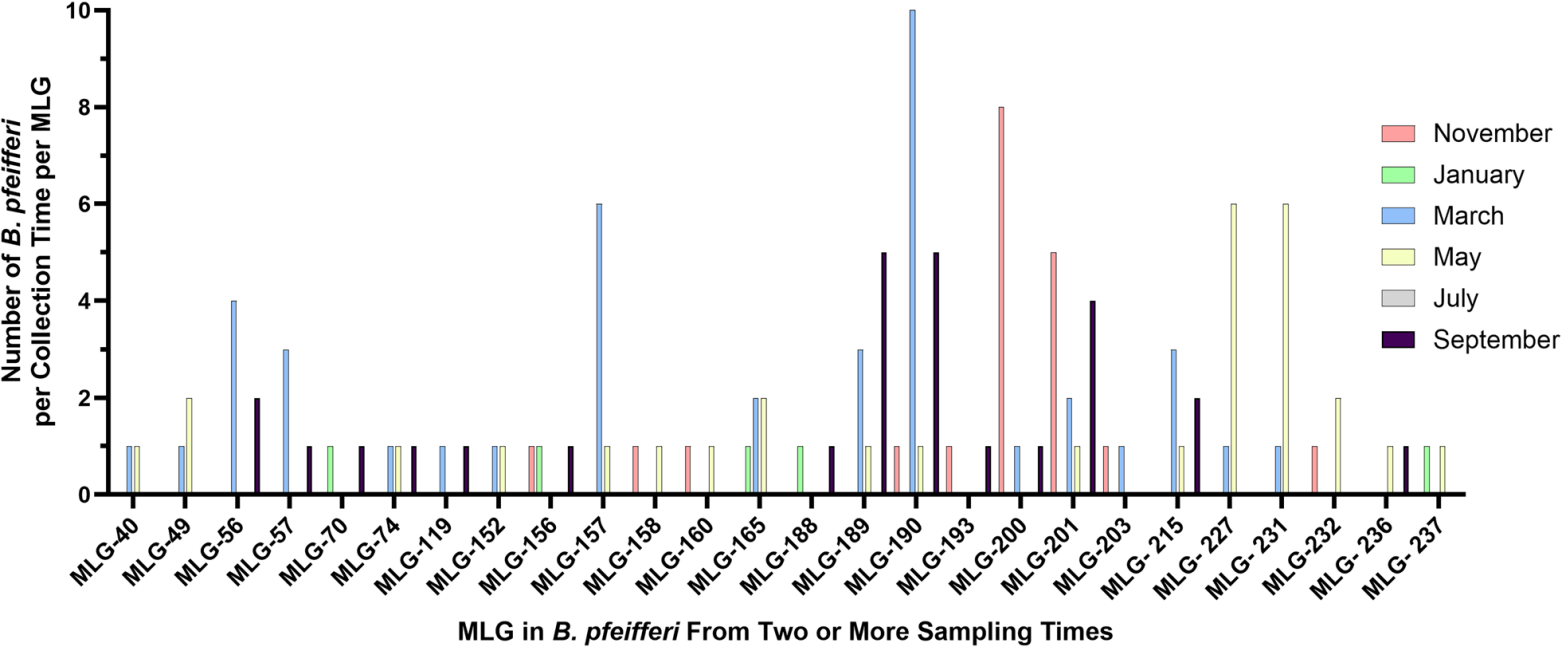
The number of *B. pfeifferi* represented, for each of 26 multilocus genotypes (MLGs) that were found at two or more of the different sampling times. Note that MLGs 190, 193, 200, and 201 were all recovered at both the first and the last sampling periods

We found that, among the 502 snails, 319 distinct MLGs were represented, 26 of which were found at two or more sampling time points. Table 3 and Fig. 3 provide overviews of the distribution of MLGs for the *B. pfeifferi* samples collected at each time point.

**Table 3:** Multilocus genotypes data of the*Biomphalaria pfeifferi* population collected from Asao Stream for each month between November 2018 and September 2019

Sampling time	MLG	MLG cst	MLG ≥ 2		MLGms
November 2018	80 [120]	09 [20]	21 [48]	22 [47]	
January 2019	39 [56]	04 [04]	17 [34]	17 [34]	
March 2019	82 [118]	19 [45]	17 [57]	10 [20]	
May 2019	67 [84]	16 [27]	11 [28]	06 [12]	
July 2019	13 [22]	00 [00]	09 [18]	09 [18]	
September 2019	74 [102]	14 [27]	19 [46]	15 [30]	

Note that the data for each time point should be considered separately; that is, if summed they would not yield integrated totals. The numbers in brackets represent number of snails represented by the MLGs. MLG, multilocus genotypes; MLG cst, multilocus genotypes found at two or more sampling times; MLG ≥ 2, multilocus genotypes represented by two or more individuals; MLG ms, multilocus genotypes shared by multiple individuals but found only at a single time point

The 26 MLGs encountered two or more times represent 124 of the 502 (24.7%) *B. pfeifferi* we collected. MLGs varied in their persistence and abundance. For instance, MLG 190 was found ten times in March and appeared at four sampling times and in 17 individuals overall (Fig. 3). Similarly, MLG 200 was observed eight times in November and was present at three sampling times. Along with MLGs 190 and 200, MLGs 193 and 201 were present in both the initial and final sampling times, demonstrating their persistence throughout the study. MLGs 40, 70, 119, 152, 158, 160, 188, 193, 203, 236, and 237 were each restricted to only two time points and had a single occurrence within each. MLGs 40, 49, 152, 157, 221, and 231 were only detected in two successive sampling points, spanning 3 months. The latter MLGs may be less prevalent and thus less frequently sampled or may truly be more transient over time. The July sample did not harbor any MLGs that were shared with other sampling times, likely a consequence of high-water levels resulting in few snails being collected at this time point.

**Fig. 4:**
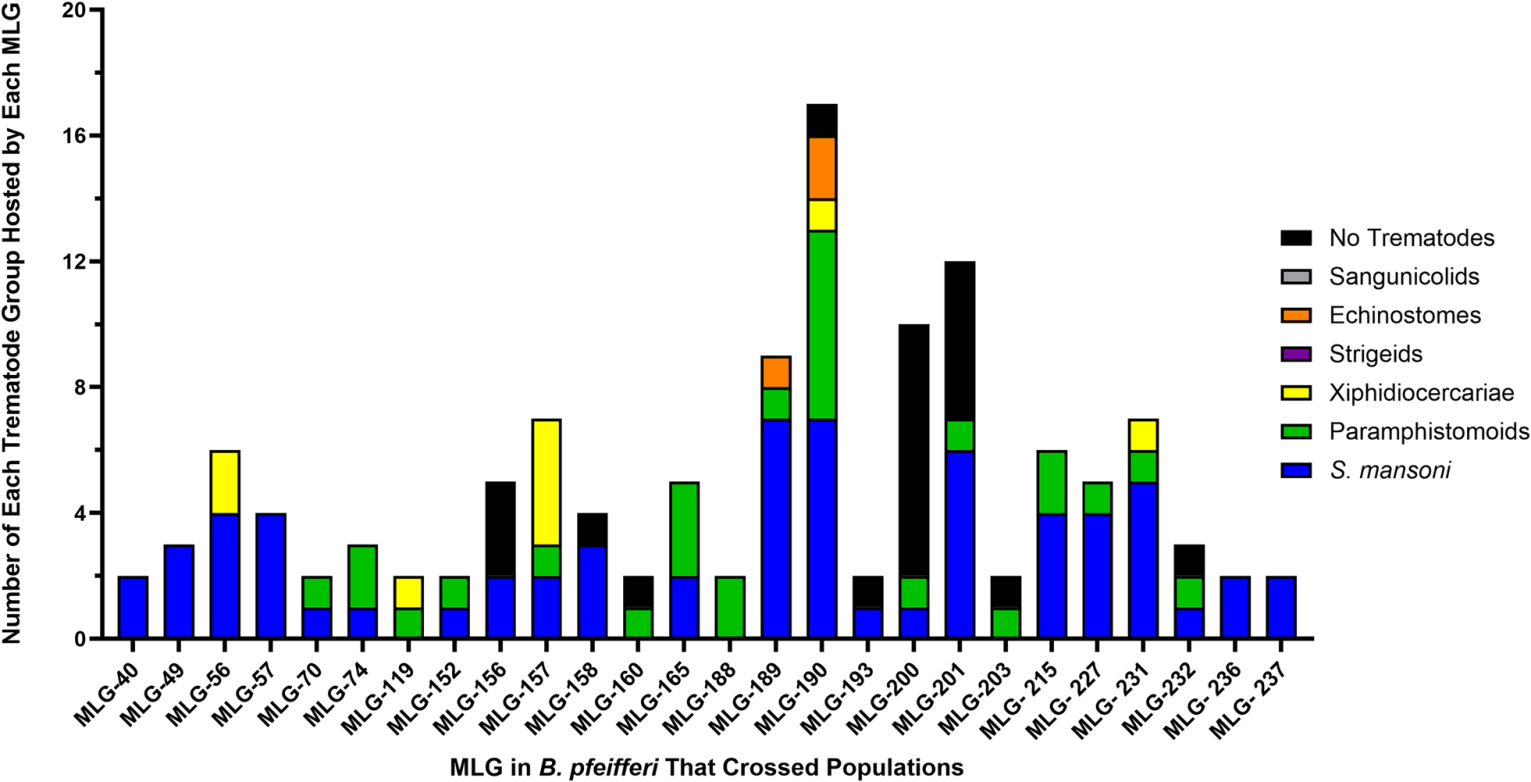
Distribution of MLG s in *B. pfeifferi* recovered from two or more sampling times and their associations with trematodes

*Schistosoma mansoni* was the most prevalent trematode species in this study and was found in 22 out of the 26 snail MLGs recovered in more than one sampling period (Fig. 4). Paramphistomoids, another prevalent group of trematodes, were present in 17 out of 26 MLGs. Eight MLGs (40, 49, 56, 156, 158, 193, 236, and 237) exhibited exclusive infection with *S. mansoni*. MLGs 160, 188, and 203 were found to harbor only paramphistomoids. MLG 190, one of the persistent MLGs identified throughout the study period, exhibited the highest number of trematode infections; 16 out of 17 snails were positive. The association between MLG 190 snails and snail infections was examined using a permutation test. We found a significant difference in the proportion of MLG 190 snails among infected snails (*p* = 0.0001), indicating that the observed distribution is unlikely to be due to random chance. A total of 103 of all 213 patent trematode infections were in the 124 snails from the repeat 26 MLGs (overall prevalence of 83.1%). The overall prevalence of all trematode infections among the *B. pfeifferi* not in MLGs found in more than one sampling time was 29.1% (110 out of 378 snails). For *S. mansoni* specifically, a total of 65 infections were from the 26 repeat MLGs (prevalence of 65 of 124; 52.4%) as compared with the overall prevalence of *S. mansoni* of 138 out of all 502 snails (27.5%) or the prevalence of *S. mansoni* among the *B. pfeifferi* not in MLGs found in more than one sampling time (73 of 378 snails; 19.3%).

Summing across all patent (shedding) trematode infections, 213 of 502 snails were positive. Table 4 provides an overview of the distribution of MLGs, for the *B. pfeifferi* samples harboring trematodes collected at each time point. The table also reveals the number of MLGs harboring trematodes that were represented at two or more sampling time points.

**Table 4:** Multilocus genotypes data of *Biomphalaria pfeifferi* harboring trematodes collected from Asao Stream

Sampling time	*N*	MLG	MLG cst
November 2018	03	03	01 [01]
January 2019	20	20	04 [04]
March 2019	90	68	32 [55]
May 2019	46	39	21 [24]
July 2019	00	00	00 [00]
September 19	54	44	17 [24]

*N* here indicates the number of individuals harboring trematodes. MLG refers to the number of multilocus genotypes harboring trematodes, MLG cst refers to the multilocus genotypes harboring trematode that were recovered from two or more sampling times. Numbers in brackets represent number of snails represented by the MLG cst. MLG, multilocus genotypes; MLG cst, multilocus genotypes found at two or more sampling times

The inbreeding coefficient (*F*_IS_) measures the degree of inbreeding within a subpopulation relative to the total population. The data in Table 5 reveal that all six sampling points exhibited high levels of inbreeding, as evidenced by positive global *F*_IS_ values. *F*_IS_ was used to estimate the degree to which selfing (*S*) contributes to the overall inbreeding in the population. According to our results, the selfing rates accounted for 77.8% (95% confidence interval [CI] 74%, 84%) of inbreeding.

**Table 5:** Inbreeding coefficient and selfing rates for samples of *Biomphalaria pfeifferi* collected at the six different time points

Sampling time	*N*	*F*_IS_	*S*
November 2018	120	0.624	0.768
January 2019	56	0.643	0.783
March 2019	118	0.517	0.682
May 2019	84	0.730	0.844
July 2019	22	0.665	0.799
September 2019	102	0.653	0.790

*F*_IS_, inbreeding coefficient; S, selfing rate

A summary of population assignment analysis for *B. pfeifferi* is shown in Table 6. For each sampling time, the analysis assigned individuals to either their own sampling time (self) or to a different sampling time (other) on the basis of their genetic profiles. The total number of individuals assigned to self and to other sampling times was 180 (36%) and 322 (64%), respectively, meaning there is considerable carryover of genotypes during the 10-month period represented by the samples. The July samples had all its individuals [22] assigned to their original sampling time, while September had only 2 out of its 102 individuals assigned to their original sampling time.

**Table 6:** Summary of the population assignment test of the 502 *Biomphalaria pfeifferi* samples

Population	Self population	Other population
November 2018	39	81
January 2019	26	30
March 2019	83	35
May 2019	8	76
July 2019	22	0
September 19	2	100
Total	180	322
Percent	36%	64%

Analysis of molecular variance for the six sampling times of *B. pfeifferi* was conducted using 999 permutations and was estimated using standard *F*-statistics showing the sources of genetic variation (Table 7). The results showed that most of the genetic variation (58%) is found among individuals within sampling times, a significant portion (33%) is found within individuals, and a smaller portion (8%) is found among sampling times. Pairwise F_ST_ values ranged from 0.008 to 0.376, with all comparisons being statistically significant (*p* < 0.05; Table S1). Low genetic differentiation was observed between November and March (F_ST_ = 0.019; *p* = 0.003). July showed the highest genetic differentiation, particularly with March (F_ST_ = 0.376; *p* = 0.001) and November (F_ST_ = 0.354; *p* = 0.001). There was no significant correlation between F_ST_ and time, as shown by weak negative correlation (*r* = −0.051; *p* =  > 0.05) revealed by the Mantel test (Fig. S1). The cumulative percentage of variation explained by the first three axes of a principal coordinates analysis (PCoA) is 97.5% (Table S2; Fig. S2).

**Table 7:** Analysis of molecular variance for the six sampling times of *Biomphalaria pfeifferi*

Source	df	SS	MS	Est. var.	Percentage (%)	*p*-Value
Among times	5	240.881	48.176	0.269	8	< 0.001
Among individuals	496	2352.463	4.743	1.843	58	< 0.001
Within individuals	502	530.500	1.057	1.057	33	< 0.001
Total	1003	3123.844		3.169	100	

Source indicates the source of the variation. Percentage is the percentage of total genetic variance attributed to the source. *df* degree of freedom, *SS* Sum of squares, *MS* mean square, *Est.Var.*estimated variance,

## Discussion

*Biomphalaria pfeifferi* supports a complex community of digenetic trematodes in Asao Stream, including a high prevalence of *S. mansoni* infections at the collection site chosen for this study [22, 43, 44], also a site of active water contact and use by the local community. The broad distribution of this highly schistosome-compatible snail across sub-Saharan Africa and South West Asia makes it, in aggregate, the world’s most important vector snail host for *S. mansoni* [2, 14].

Here, we show that *B. pfeifferi* from Asao had an excess of homozygotes, as demonstrated by the highly significant global *F*_IS_ (Table 5), with heterozygosity values for all sampling times being low. The low levels of genetic variability evidenced by AMOVA and the population assignment test in this study are similar to the findings of studies on genetic diversity in *B. pfeifferi* populations from Madagascar using the same markers [45]. One of the most intriguing aspects of the biology of *B. pfeifferi* is that it is a predominantly self-fertilizing species [8], with attendant consequences for its population structure. We also confirm using *F*_IS_ that *B. pfeifferi* from Asao are selfers, with selfing rates as high as 77%. We observed weak differentiation among samples from different time points, as they exhibited low values of F_ST_ (as low as 0.010). This is not surprising given that all samples were drawn from the same location but at different sampling times. These values of F_ST_ were lower than the values reported by Charbonnel et al. [45] at a similar geographic location, and consistent with values reported in other selfing snail species noted by Viard et al. [46]

Owing to its high selfing rate, individual *B. pfeifferi* might give rise to distinctive MLGs that are then maintained across generations by repeated selfing, and that the aggregate *B. pfeifferi* population in a given area might consist of multiple MLGs that potentially differ in their susceptibility to *S. mansoni* and other parasites. Previous population studies of *B. pfeifferi* have not explicitly examined the notion that identifiable MLGs lineages comprised of individuals with largely similar genotypes exist within *B. pfeifferi* populations can be followed over time, and their status with respect to hosting trematodes compared with individuals in other MLGs from the same locality.

The present study provides an initial attempt to explore in six bimonthly samples over a 10-month span the genetic constitution with respect to MLGs of a single *B. pfeifferi* population known to bear a heavy parasite burden. The overall prevalence of larval digeneans infections among the 502 *B. pfeifferi* examined at our single Asao collecting site was indeed high—42.4%—with over half of the infections recorded being of the medically important trematode *S. mansoni* (138 of 502 snails; a prevalence 27.5%). We acknowledge that some of the infections designated here as *S. mansoni* might in fact be of the closely related and morphologically similar rodent parasite *S. rodhaini*, but in the materials and methods section we explain why that proportion is likely to be low. Although molecular identification of all the schistosome infections would be desirable, it was beyond the scope of the present study.

Using 14 codominant microsatellite loci and employing clone correction, we identified 319 unique MLGs among our *B. pfeifferi* samples. Although more extensive sampling over longer periods would be desirable, our study indicates that recognizable MLGs can be identified and that some persisted over the 10 months of the study. The presence of particular MLGs, such as MLG 190, 193, 200, and 201, over 10 months indicates their ability to persist in varying environmental conditions. Although *B. pfeifferi* can survive in the laboratory for as long as 100 weeks [47] and laboratory survival of individuals infected with *S. mansoni* has been recorded for over a year [48], average life expectancy in the field is considerably lower, 2–3 weeks [49]. If this is so, the 10-month duration of our study may have been long enough to have captured individuals with the same MLG but representing different generations.

Furthermore, we found intriguing indications to hint that the more common or persistent MLGs are prone to trematode infection, supporting both a high prevalence of infection and diverse trematodes. For 26 MLGs that were recovered at two or more sampling times, in aggregate representing 124 snails, the prevalence of infection for all trematodes was 103 of 124 snails (83.1%), in contrast to the prevalence in snails with an MLG found in only one sampling time or that had unique genotypes not found in other snails, which was 110 of 378 snails (29.1%). Furthermore, the prevalence of *S. mansoni* among the snails representing the 26 MLGs found more than once was higher (65 of 124; 52.4%) as compared with snails with an MLG found at only one sampling time or that had unique genotypes (73 of 378; 19.3%). Similarly, for the most commonly recovered MLG, MLG 190—one that persisted throughout the 10-month study—16 of 17 (94.1%) snails were positive for trematode infection, including the broadest representation of trematode diversity seen for any MLG; additionally, 7 of these 17 (41.2%) were positive for *S. mansoni*. These results highlight the potential existence of persistent *B. pfeifferi* MLGs prone to transmitting a diverse range of trematodes, including *S. mansoni* [44, 50, 51].

With respect to thoughts that some *B. pfeifferi* genotypes might be found that are refractory to *S. mansoni*, 4 of the 26 MLGs found more than once were not found to harbor *S. mansoni*, though they were not among the most common repeat MLGs found. Furthermore, although the prevalence of *S. mansoni* infection among MLGs found only once or in snails with unique genotypes was considerably lower (19.3%) than the 52.4% prevalence in MLGs found more than once, the former group comprised, in aggregate, more *S. mansoni* infections (*n* = 73) than the latter (*n* = 65). Infection rates for different MLGs may be influenced by inherent differences in resistance, longevity (with longer-lived MLGs eventually accumulating more infections), or post-infection survivorship. Trematode infection imposes considerable fitness costs on infected snails, which are typically castrated and suffer higher mortality rates than uninfected snails [52].

Red Queen dynamics are known to occur in freshwater snails capable of selfing and exposed to heavy trematode burdens. In the freshwater snail *Potamopyrgus antipodarum*, diploid individuals capable of sexual reproduction and triploid females that reproduce by parthenogenesis might be present, the latter resulting in production of distinct clones of snails. Snails within a clone are capable of rapid population expansion (all individuals can produce progeny), but the genetic homogeneity within clones potentially renders them vulnerable to attack by parasites, more so than progeny produced by sexual reproduction, which differ genetically from one another [53]. Progeny resulting from sexual reproduction might harbor unique combinations of resistance genes, which might diminish parasite success rates. In its native lacustrine habitats, given that trematode pressure is highest around lakeshores as compared with deeper water, *P. antipodarum* tends to engage in sexual reproduction in shoreline habitats, whereas snails from deeper waters, where parasites pressures are less, tend to reproduce parthenogenetically[54].

These results suggests that basic Red Queen dynamics as exemplified by the *P. antipodarum* might exist in *B. pfeifferi* as well, though much more study is required to learn whether common or long-standing MLGs come to have higher trematode loads than individuals from rare or newly appearing MLGs. If so, this may be because they become more susceptible over time as trematodes adapt to them, or it is possible they inherently live longer and so have higher chances of acquiring trematode infections. It should not be forgotten that *B. pfeifferi* is capable of cross-fertilization [7, 9], and the frequency with which this might occur in habitats such as Asao is not known. Unlike the New Zealand Lake situation, however, repeated episodes of seasonal flooding followed by periods of drought occur at Asao Stream, though it has not been observed to completely dry out since 32 years ago, when it was initially identified as a *B. pfeifferi* habitat where *S. mansoni* transmission took place [21]. Flooding may fundamentally reset the situation in Asao with respect to MLG representation, thereby preventing Red Queen dynamics from ever becoming sufficiently strong to favor sexual reproduction. What is clear though is that the *B. pfeifferi* population at Asao remains remarkably robust across many years, in spite of the high levels of trematode parasitism exhibited.

## Conclusions

This study reports the presence of identifiable MLGs within a natural population of *B. pfeifferi* in Asao Stream, western Kenya, some of which persisted over the 10-month duration of the study. The study also highlights an association of particular MLGs with the transmission of *S. mansoni* and other trematodes. New approaches to schistosomiasis control targeting snail vectors such as *B. pfeifferi* are needed, as traditional methods such as use of molluscicides are expensive and often environmentally unacceptable [55]. Identifying and exploiting traits such as snail resistance to schistosome infection offers one way forward [56, 57]. Although *B. pfeifferi* is generally considered to be a species highly susceptible to *S. mansoni* infection [48], among the several intrapopulation MLGs that comprise natural *B. pfeifferi* populations, we may be overlooking particular MLG variants that can be shown to be persistently resistant to *S. mansoni*. Identification of the underlying traits responsible for schistosome resistance might be much more possible using this route rather than traditional approaches based on laboratory-reared snail populations. This is because *B. pfeifferi* laboratory colonies are difficult to maintain and tend to rapidly lose their inherent variability. Identification and manipulation of such resistant MLG variants may prove useful in intervention strategies to control schistosomiasis in Asao Stream and similar African ecosystems.

## Supplementary Information

Additional file 1.

## Data Availability

The data supporting the findings of the study must be available within the article and/or its supplementary materials, or deposited in a publicly available database.

## Abbreviations

MLG: Multilocus genotypes
AMOVA: Analysis of molecular variance
PCR: Polymerase chain reaction
EDTA: Ethylenediaminetetraacetic acid
Ho: Observed heterozygosity
*F*_IS_: Fixation index within subpopulations (inbreeding coefficient among subpopulations)
F_ST_: Fixation index among subpopulations
KEMRI: Kenya Medical Research Institute
