# Supplementary material for: Evidence for persistent multilocus genotypes of Biomphalaria pfeifferi in a natural population in Kenya, with implications for transmission of Schistosoma mansoni
Source: Parasit Vectors. 2025 Jun 21;18:235. doi: 10.1186/s13071-025-06881-1 (PMC12182688; doi:10.1186/s13071-025-06881-1)
Supplement: Supplementary file 1 — Additional file 1. [file 13071_2025_6881_MOESM1_ESM.docx]

**Supplementary material**

**Table S1*:* Pairwise Fst Analysis of *B pfeifferi* per sampling time.**

|  | **Nov-18** | **Jan-19** | **Mar-19** | **May-19** | **Jul-19** | **Sep-19** |
| --- | --- | --- | --- | --- | --- | --- |
| **Nov-18** |  | 0.001 | 0.003 | 0.001 | 0.001 | 0.001 |
| **Jan-19** | 0.117 |  | 0.001 | 0.001 | 0.001 | 0.001 |
| **Mar-19** | 0.019 | 0.157 |  | 0.001 | 0.001 | 0.001 |
| **May-19** | 0.047 | 0.051 | 0.064 |  | 0.001 | 0.031 |
| **Jul-19** | 0.354 | 0.166 | 0.372 | 0.222 |  | 0.001 |
| **Sep-19** | 0.050 | 0.046 | 0.052 | 0.008 | 0.184 |  |

**F_ST_ values below diagonal, P values above diagonal.**

**Figure S1: Correlation Between Fst and Temprol time of *B pfeifferi***

**Table S2: Principles of Coordinates Results for *Biomphalaria Pfeifferi.***

| **Percentage of variation explained by the first 3 axes** | | | | |
| --- | --- | --- | --- | --- |
|  |  |  |  |  |
| **Axis** | **1** | **2** | **3** |  |
| **%** | 77.93 | 14.71 | 4.81 |  |
| **Cum %** | 77.93 | 92.64 | 97.45 |  |
|  |  |  |  |  |

**Figure S2: Principal Coordinates (PCoA)**

The percentage of variation explained by the first three axes of a principal coordinates analysis (PCoA), was determined for *B. pfeifferi* sampled (supplementary table 2, supplementary 2). The data show that the first axis explains a large proportion of the variation in the dataset (77.93%), while the second and third axis explain smaller proportions (14.71% and 4.81%, respectively). The cumulative percentage of variation explained by the first three axes is 97.45%.
